# Case Report with Literature Review: Tumor-induced osteomalacia from a soft-tissue phosphaturic mesenchymal tumor of the trunk

**DOI:** 10.3389/fendo.2025.1655376

**Published:** 2025-09-18

**Authors:** Huiyuan Tao, Zhimin Deng, Li Chen, Wenli Wang, Yuqing Zhou, Yue Wu

**Affiliations:** ^1^ Dongguan Hospital of Guangzhou University of Chinese Medicine, Dongguan, Guangdong, China; ^2^ Department of Endocrinology, Dongguan Hospital of Guangzhou University of Chinese Medicine, Dongguan, Guangdong, China

**Keywords:** tumor-induced osteomalacia, phosphaturic mesenchymal tumor, hypophosphatemia, soft-tissue, trunk

## Abstract

**Background:**

Tumor-induced osteomalacia (TIO), a type of acquired hypophosphatemic osteomalacia, is brought on by tumors producing excessive levels of fibroblast growth factor 23, which raises renal phosphorus excretion.

**Methods:**

Through a review of the literature, we have outlined the clinical characteristics of 33 patients with soft-tissue TIO of the trunk and described a case of TIO brought on by a soft-tissue tumor on the back.

**Results:**

A 63-year-old woman who had been experiencing generalized bone pain for approximately three years visited the hospital. Physical examination revealed a round mass on the back measuring approximately 2 × 2 cm. Laboratory tests showed low blood phosphorus, elevated synchronous urinary phosphorus, and elevated alkaline phosphatase levels. The mass was detected using magnetic resonance imaging and ultrasound, and it was subsequently surgically excised. Following surgery, phosphate levels returned to normal, bone pain was relieved, and pathology confirmed phosphaturic mesenchymal tumor (PMT). A literature review identified only 33 cases of soft-tissue TIO occurring in the trunk, with a mean age of 49.7 ± 15.6 years and a male-to-female ratio of 23:10. Bone pain was present in 91% of patients, and diagnostic delay of more than two years was observed in 72.4% of cases. The mean preoperative serum phosphorus level was 0.48 ± 0.137 mmol/L, and the median tumor size was 3 cm (IQR: 2–4.65 cm). Postoperative remission of biochemical indices and clinical symptoms was observed in 96.9% of patients, with no recurrence during the follow-up period. The majority of tumors (72.7%) were pathologically diagnosed as PMT.

**Conclusion:**

Soft-tissue TIO of the trunk is rare. Clinicians should be alert to the possibility of TIO in patients with unexplained bone pain and hypophosphatemia and should promptly perform appropriate examinations to avoid missed diagnoses.

## 1 Introduction

Tumor-induced osteomalacia (TIO) is a type of acquired hypophosphatemic osteomalacia caused by excessive production of fibroblast growth factor 23 (FGF23) by tumors, leading to decreased renal reabsorption and increased urinary excretion of phosphorus. Clinically, patients present with symptoms such as weakness, bone pain, bone deformities, and multiple fractures. Once the tumor is removed, blood FGF23 levels decrease, blood phosphorus levels rise, and the condition improves markedly, with most patients achieving complete recovery. Although tumors causing TIO can occur in any part of the body, soft-tissue tumors of the trunk are rarely reported. Therefore, we present a case of TIO brought on by a phosphaturic mesenchymal tumor (PMT) in the soft tissues of the back and conduct an extensive review of the relevant published research.

## 2 Materials and methods

### 2.1 Case description

A 63-year-old woman was admitted to our department on June 10, 2021, with generalized bone pain that had persisted for approximately three years. The patient had sustained a right ankle fracture three years earlier following an accidental fall, which healed with conservative treatment. She developed generalized bone pain, which was initially present in the lower back before gradually spreading to the limbs. She was diagnosed with severe osteoporosis at several hospitals. She received treatment with oral alendronate as well as calcium and vitamin D supplementation, but the therapeutic outcomes were unsatisfactory. The pain gradually intensified, accompanied by fatigue. The symptoms began with weakness in both lower limbs, gradually developing into general weakness. She experienced increasing difficulty in getting up and turning over, and a wheelchair was needed for movement. She underwent lumbar internal fixation surgery for lumbar disc herniation at other hospitals more than a year earlier and was later admitted to our outpatient clinic in February with a diagnosis of bilateral rib fractures. She was managed conservatively at our hospital as an outpatient. She had no history of hypertension or diabetes mellitus and denied any family history of hereditary or infectious diseases, such as hepatitis. She had been menopausal for nearly 10 years. Physical examination revealed a rounded, soft, non-indurated, and movable mass on the back, measuring approximately 2 × 2 cm. There was tenderness in the spinous processes of the lumbar vertebrae. There was no deformity in the pelvis, but there was mild squeezing pain. A thoracic compression test (+) was conducted. The distance from the costal margin to the iliac spine was approximately 5–6 cm. We performed auscultation of the heart and lungs (-) and abdominal physical examination (-). The muscle strength of both upper limbs was grade V, and that of both lower limbs was grade IV. Physiological reflexes were present, but pathological signs were not elicited.

Laboratory tests upon admission and their results were: biochemical tests: blood, urine, and stool routine tests were normal. Blood phosphorus: <0.30 mmol/L, blood calcium: 2.10 mmol/L, 24-h urine phosphorus 15.3 mmol (synchronous blood phosphorus <0.3 mmol/L), 24-h urine calcium 2.31 mmol (synchronous blood calcium 2.10 mmol/L), alkaline phosphatase 265 IU/L (see **Table 1**), alanine aminotransferase 11 IU/L (7–40 IU/L), blood glucose 5.68 mmol/l (3.6–6.1 mmol/L), creatinine 56.6 µmol/L; blood gas analysis: blood pH was 7.426 (7.35–7.45), partial pressure of carbon dioxide was 4.38 kPa (4.26–5.99 kPa), and concentration of bicarbonate (cHCO_3_-) was 21.2 mmol/L. Endocrine hormones: parathyroid hormone 10.4 pmol/L (synchronous blood calcium 2.31 mmol), growth hormone 0.256 ng/mL. The cortisol rhythm, insulin-like growth factor 1 (IGF-1), and thyroid function were all normal. Bone metabolism indicators: total vitamin D 12.87 ng/mL, β-collagen degradation products 0.570 ng/mL, total type I collagen amino-terminal elongated peptide 169.5 ng/mL, osteocalcin 14.36 ng/mL (7.7–21.7 ng/mL), and calcitonin 0.50 pg/mL.

**Table 1:** Some laboratory indicators and reference ranges at admission.

Inspection items	Result	Reference range
Serum phosphorus	<0.30	0.85–1.51 mmol/L
24-h urine phosphorus	15.3	mmol/24 h
Serum calcium	2.10	2.11–2.52 mmol/L
24-h urinary calcium	2.31	mmol/24 h
Parathyroid hormone	10.40	1.60–6.90 pmol/L
Total vitamin D	12.87	ng/mL
Alkaline phosphatase	265	50–135 IU/L
TmP/GFR	0.16	0.80–1.35 mmol/L

TmP/GFR, tubular maximum reabsorption of phosphate per glomerular filtration rate.

**Figure 1:**
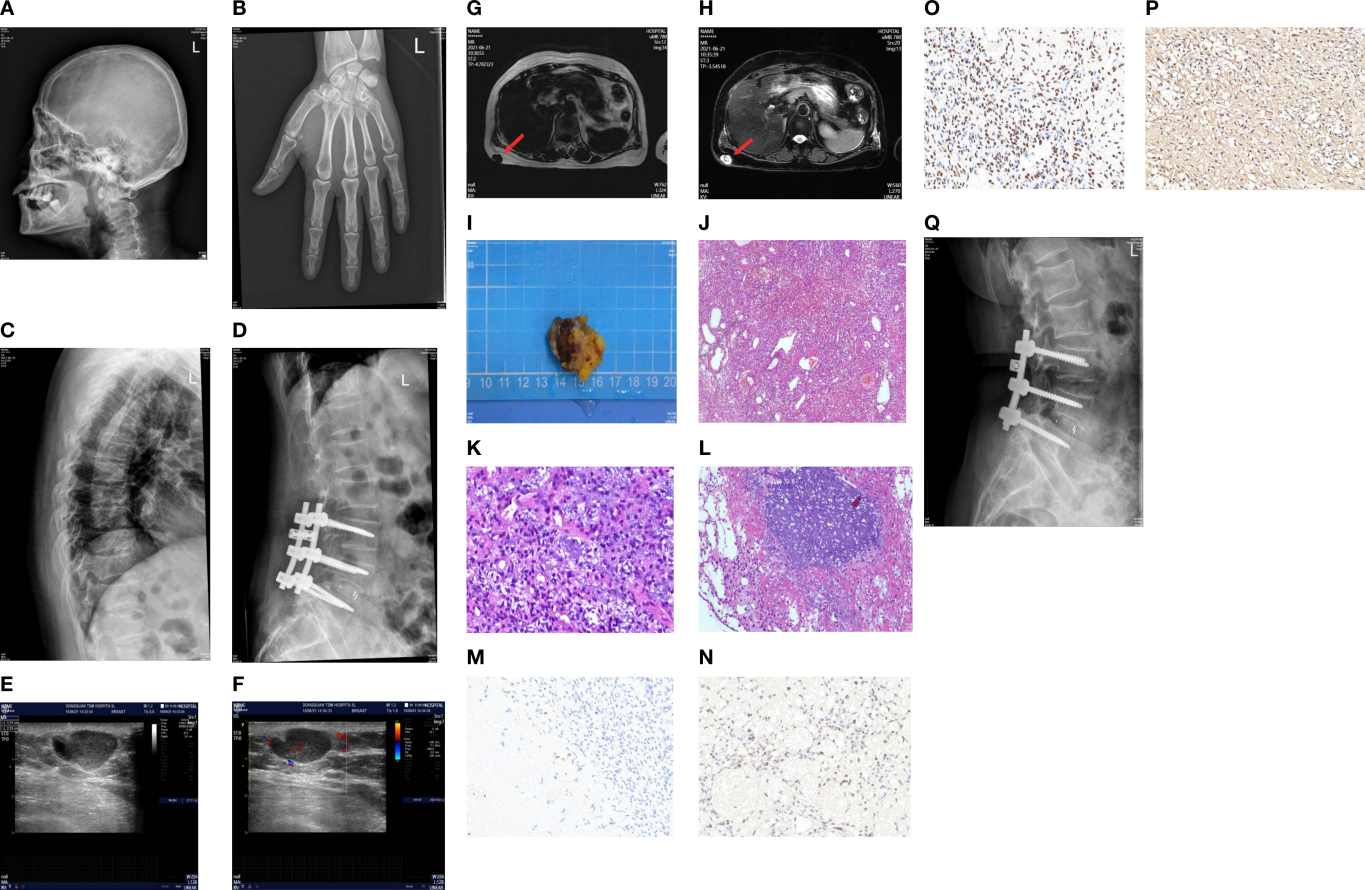
**(A)** Left side of the skull on digital radiography (DR). **(B)** Orthostatic X-ray image of the left hand on DR. **(C, D)** DR thoracolumbar lateral film. **(E, F)** Ultrasound showed a hypoechoic mass in the subcutaneous fat layer of the right back, measuring approximately 23 × 12 × 20 mm in size, with a clear boundary. The mass was mainly internally hypoechoic and was surrounded by a liquid dark area. CDFI: Short linear blood flow signals can be seen in the periphery and inside. **(G, H)** 3.0T MRI chest plain scan: A nodular abnormal signal shadow was observed in the subcutaneous fat layer of the right back, approximately 14 × 21 mm in size. T1W1 showed a relatively low signal intensity, while T2W1 and fat compression showed a mixed high and low signal intensity. The shape was irregular and lobulated, closely adhering to the adjacent skin, with a clear and smooth residual edge. **(I)** The tumor was a soft gray-brown tissue, 3.0 × 3.0 × 2.0 cm in size. **(J–L)** Microscopic observation: The submitted tissue was composed of proliferating blood vessels and spindle cells, with unclear boundaries from the surrounding tissues. Blue-stained granular interstitial substances (stained with hematoxylin and eosin) could be seen between the spindle cells. Immunohistochemistry showed spore cells, Vim positivity, CD56 positivity, CD34 negativity, and Ki-67 positivity with a hotspot area of approximately 10%. **(M–P)** Immunohistochemical staining of FGF23, Ki67, SATB2, and SSTR2A (40×). **(Q)** The lateral lumbar spine X-ray of the patient 6 months after the operation.

Imaging studies showed generalized abnormalities of the skeletal system. The bone mineral density of the orthotopic lumbar vertebrae (L1–L4) was 0.716 g/cm^2^, with a T-score of -3.3. A whole-body digital radiography (DR) scan showed degenerative changes in the pelvis; degenerative changes and osteoporosis in the lumbar spine; decreased bone mineral density of the thoracic and lumbar vertebrae with varying degrees of vertebral body flattening; and the presence of the “double-concave sign” in some vertebral bodies. Post-fixation changes were observed in the lumbar vertebrae (L3–L5), along with osteoporosis of the cranial vault bones and both metacarpals (**Figures 1A–D**).

Ultrasound of the mass on the back showed a hyperechoic lesion in the subcutaneous fat layer measuring approximately 23 × 12 × 20 mm, with clear boundaries and predominantly hyperechoic internal echoes, although its nature remained undetermined (**Figures 1E, F**). Magnetic resonance imaging (MRI) of the chest revealed a nodular abnormal signal shadow in the subcutaneous fat layer on the right side of the back, measuring approximately 14 × 21 mm, with a relatively low signal intensity on T1-weighted images and mixed high and low signal intensity on T2-weighted images and fat-suppressed sequences. The mass exhibited an irregular, lobulated morphology and was closely adherent to the adjacent skin, with the remaining edges appearing clear and smooth (**Figures 1G, H**).

The patient has been diagnosed with osteoporosis multiple times in the past. However, generalized bone pain, fatigue, and decreased muscle strength were evident during physical examination, which do not match the clinical manifestations of osteoporosis. The patient’s laboratory tests showed significant hypophosphatemia, a marked increase in synchronous urinary phosphorus, and elevated alkaline phosphatase, none of which support the diagnosis of osteoporosis. Whole-body DR scans of some vertebrae revealed double concave changes, and lateral head films showed blurred trabeculae and other manifestations of osteomalacia, rather than characteristic changes of osteoporosis. The patient’s age of onset, clinical manifestations, laboratory tests, and DR manifestations supported the diagnosis of hypophosphate-osteomalacia in metabolic bone diseases and ruled out metabolic bone diseases caused by other diseases such as multiple myeloma, hypercortisolism, and hyperparathyroidism. The patient was middle-aged or older with no family history, and hereditary hypophosphatemic osteomalacia was not considered. If acidosis is absent, then hypophosphatemic osteomalacia caused by renal tubular acidosis should not be considered. Based on a comprehensive analysis, tumor-related hypophosphatemic osteomalacia was considered.

A mass can be palpated in the subcutaneous fat layer on the patient’s back. Given the superficial location of the mass, a localized lumpectomy was performed on June 23, 2021. During the operation, a transverse, shuttle-shaped incision approximately 4 cm in length was made along the dermatoglyphics, centered over the right back mass. The skin was incised to expose the mass, which was soft and well-defined. The mass, along with the surrounding fatty tissue, was excised using an electrocautery device, and the specimen was sent for pathological examination. Visual inspection of the excised tissue indicated a piece of grayish-brown tissue, 3.0 cm × 3.0 cm × 2.0 cm in size, and soft in texture. Microscopic observation: The resected tissue comprised proliferating blood vessels and spindle cells, with an indistinct boundary from the surrounding tissues. Blue-stained granular interstitium was seen between the spindle cells.

Postoperative pathology revealed the lesion to be consistent with a PMT (**Figures 1I–L**). Immunohistochemical staining demonstrated spindle cell Vim expression, neural cell adhesion molecule (CD56) expression, lack of cluster of differentiation 34 (CD34) expression, a Kiel antigen 67 (Ki-67) hotspot area of approximately 10%, as well as expression of FGF23, growth inhibitory receptor 2A (SSTR2A), and specialized AT-rich sequence-binding protein 2 (SATB2) (**Figures 1M–P**).

**Figure 2:**
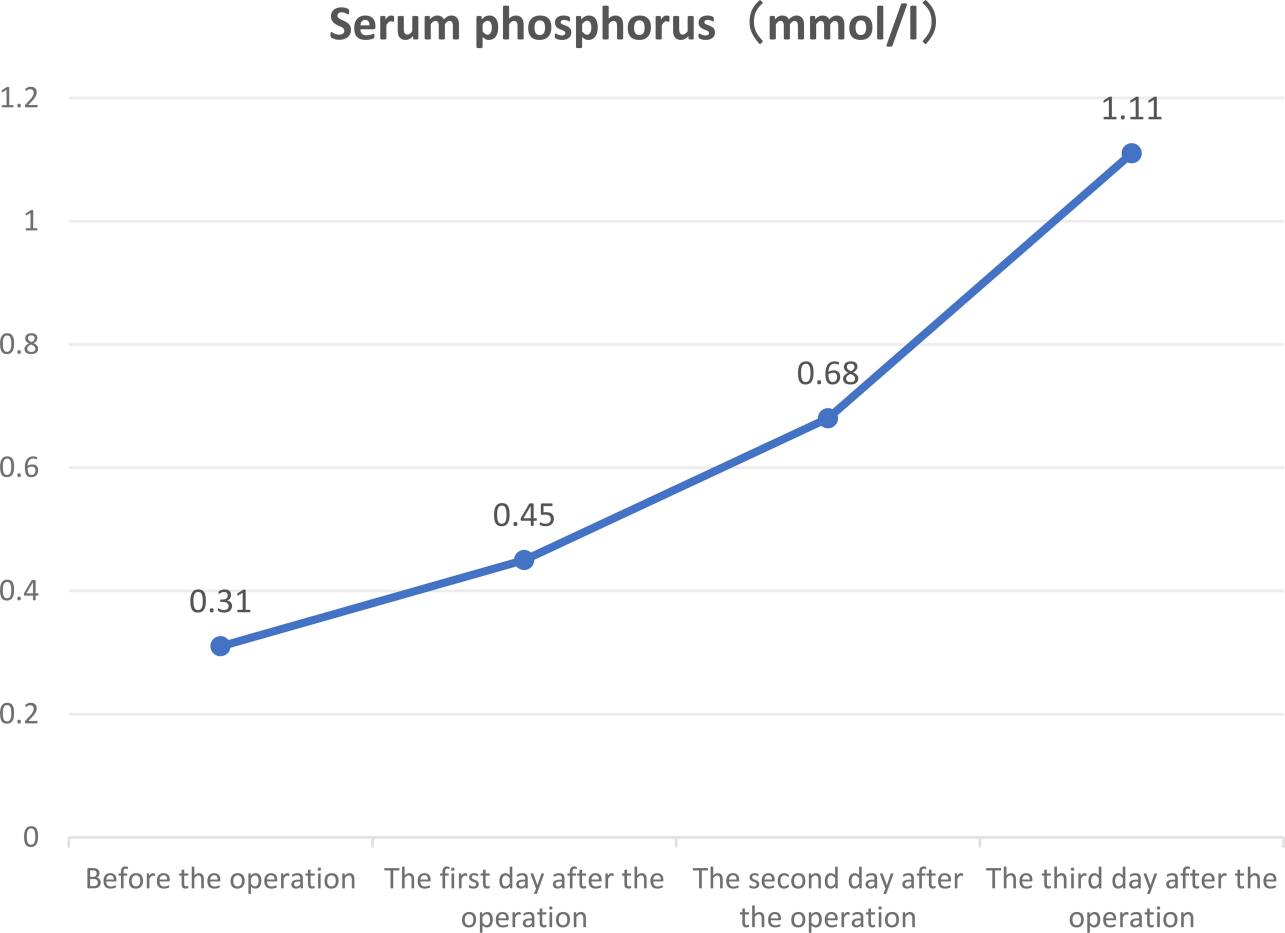
Three-day trend chart of changes in preoperative and postoperative serum phosphorus in patients.

Blood phosphorus levels were assessed on the first postoperative day at 0.45 mmol/L (reference range: 0.85–1.51 mmol/L), on the second day at 0.68 mmol/L, and on the third postoperative day at 1.11 mmol/L, returning to normal levels (**Figure 2**). Six weeks after the operation, the blood phosphorus level was 1.51 mmol/L, and the urine phosphorus level was 8.2 mmol/24 h. The patient reported that the pain and fatigue throughout her body had significantly lessened compared to before. Considering that patients with TIO may develop bone starvation syndrome following an operation and that our patient was an older postmenopausal woman (1, 2), 0.6 g of calcium carbonate D3 (caltrate D) and 0.25 µg of calcitriol capsules were continuously administered after the operation. The patient’s pain and fatigue symptoms were basically relieved, and she could walk independently one month after the operation. Six months after the operation, the lateral DR lumbar spine X-ray was reexamined, and the cortical bone was thicker than that before the operation (**Figure 1Q**).

The study received approval from our hospital’s institutional review board, and because of its retrospective nature, formal informed consent was not required from the patient.

### 2.2 Literature review

Soft-tissue TIO of the trunk includes TIO occurring in the chest, lungs, abdomen, breasts, and back (excluding the spine and ribs). A PubMed database search was conducted using the terms “tumor-induced osteomalacia,” “phosphaturic mesenchymal tumor,” and “oncogenic osteomalacia,” covering literature up to January 1, 2025. A total of 33 cases of soft-tissue TIO of the trunk were identified, with detailed information presented in **Table 2**.

**Table 2:** Literature review of 33 cases of soft tissue TIO in the trunk.

Authors	Year	Age & Sex	Symptom	Diagnostic delay time (Y)	Serum phosphorus	Diagnostic method	Location	Tumor size	Treatment	Result	Follow-up time (M)	Histological diagnosis	Immunohistochemistry
Chen et al. (3)	2020	54 M	Stress fracture, bilateral hip pain	7	1.3 mg/dL (2.7–4.6 mg/dL)	PET-CT, MRI	Soft tissue of left scapula	3.9 × 3.3 × 4.3 cm	Surgical resection	Complete resolution	4	PMTMCT	NA
DeWitt et al. (4)	2007	52 M	Diffuse pain, progressive weakness, loss of height	5	1.8 mg/dL (2.5–4.8 mg/dL)	PET-CT, clinical examination	Left posterior back nodule	1.5 cm	Surgical resection	Complete resolution	6	PMTMCT	NA
Gardner et al. (5)	2013	40 M	Progressive pain in legs, lower back, and ribs	4	0.58 mmol/L (0.81–1.45 mmol/L)	Clinical examination, 18F-FDG PET/CT	Subcutaneous nodule on left shoulder	2.0 × 1.7 cm	Surgical resection	Complete resolution	5	PMTMCT	CD34 positive
Graham et al. (6)	2012	47 M	Bone pain	8	NA	MRI	Suprascapular tangential mass	3.8 cm	Surgical resection	Complete resolution	NA	PMTMCT	NA
Gu et al. (7)	2023	60 M	Pain in left leg	0.5	1.7 mg/dL (2.5–4.5 mg/dL)	68 Ga-DOTATATE PET-CT, MRI, CT	Mass in scapula	2.9 × 0.6 × 2.3 cm	Surgical resection	Complete resolution	12	PMT	NA
Habbsa et al. (8)	2023	34 M	Pain in shoulders, hips, ankles, and feet	6	1 mg/dL (2.8–4.1 mg/dL)	68 Ga-DOTATATE PET-CT, physical examination	Mass beside left spinal cord	8 × 2.5 cm	Surgical resection	Complete resolution	3	PMT	NA
Habra et al. (9)	2008	84 M	Generalized muscle pain and fatigue	11	1.8 mg/dL (2.5–4.5 mg/dL)	PET-CT, physical examination	Near posterior of 10th rib on left	4 cm	Surgical resection	Complete resolution	NA	Hemangiopericytoma	NA
Karaa-Zbidi et al. (10)	2016	41 M	Lower limb pain, functional impotence	1.5	0.31 mmol/L	Ultrasound, MRI, physical examination	Lateral and dorsal sides of left scapula	5 cm	Surgical resection	Complete resolution	3	Low-grade malignant osteosarcoma	NA
Kaul et al. (11)	2007	22 F	Low back pain radiating to buttocks and legs	1	2 mg/dL (3–4 mg/dL)	MRI, physical examination	Subcutaneous mass on right shoulder	2 cm	Surgical resection	Complete resolution	NA	PMT	NA
Kawai et al. (12)	2017	53 F	Bone pain in limbs, ribs, and buttocks	0.5	1.6 mg/dL (2.5–4.5 mg/dL)	68Ga-DOTATOC PET/CT, MRI	Nodule on dorsal side of right scapula	1.0 × 0.4 cm	Surgical resection	Complete resolution	6	PMTMCT	Positive for SSTR2A
Zalewska et al. (13)	2022	50 F	Bone pain, general fatigue and, muscle weakness	3	1.6 mg/dL (2.3–4.7 mg/mL)	Physical examination, ultrasound	Subcutaneous mass in right scapula area	2.7 × 2.1 × 0.6 cm	Surgical resection	Complete resolution	45	PMT	NA
Zura et al. (14)	1999	46 M	Right groin and leg pain accompanied by bilateral heel pain and muscle weakness	2.5	1.0 mg/dL (2.4-4.5 mg/dL)	Physical examination, whole-body bone scan, PET-CT	Soft tissue mass of right rib	14.3 × 14.2 × 10.4 cm	Surgical resection	Complete resolution	12	Mesenchymal chondrosarcoma	NA
Meng & Wagar (15)	2014	79 M	Progressive bone pain, muscle weakness, and height reduction	3	1.2 mg/dL (2.5–4.5 mg/dL)	CT, MRI	Soft tissue mass at 10th rib on left posterior side	8 × 4 cm	Surgical resection	Complete resolution	2	Hemangiopericytoma	FGF23 positive
Mennetrey et al. (16)	2021	66 M	Bone and joint pain and weight loss	1	0.42 mmol/L	68Ga-DOTATOC PET/CT, MRI	Left scalene muscle	3.0 × 1.1 cm	Surgical resection	Complete resolution	2	PMT	NA
Oka et al. (17)	2007	47 M	Low back and bone pain	NA	1.2 mg/dL (1.4–4.5 mg/dL)	Physical examination	Subcutaneous nodule on right back	2.8 × 1.0 × 1.0 cm	Surgical resection	Complete resolution	2.5	PMTMCT	Vimentin positive
Oyama et al. (18)	2020	13 F	Pain and fractures in both lower extremities	5	1.7 mg/dL (3.7–5.8 mg/dL)	CT, physical examination	Small mass in left trapezius muscle	1 cm	Surgical resection	Malignant transformation, multiple metastases, and death at the age of 18	48	PMTMCT transforms into pleomorphic sarcoma	FGF23 and P53 positive
Radaideh et al. (19)	2009	39 M	Diffuse bone pain, muscle weakness, and difficulty walking	3	0.6 mmol/L (0.81–1.6 mmol/L)	CT	Soft tissue mass on inner side of left psoas major muscle	5 × 1.2 × 1 cm	Surgical resection	Complete resolution	36	PMTMCT	NA
Shi et al. (20)	2018	52 F	Left posterior chest pain	10	0.38 mmol/L (0.81–1.55 mmol/L)	Tc bone scan, CT, MRI, physical examination	Left posterior chest wall	14 x 9 x 8 cm	Surgical resection	Complete resolution	8	PMTMCT	NA
Shi et al. (20)	2018	59 F	Spasm of left thoracic muscles accompanied by pain	1	0.52 mmol/L (0.81–1.55 mmol/L)	Tc bone scan, CT, MRI	Left posterior chest wall	1.5 x 1.5 x 0.5 cm	Surgical resection	Complete resolution	8	PMTMCT	NA
McHan and Augustine (21)	2024	43 M	Progressive hip pain, fatigue and weakness, and multiple incomplete fractures	5	1.3 mg/dL (2.7–4.5 mg/dL)	68Ga-DOTATATE PET/CT	Subcutaneous mass in left hypochondrium	3.5 cm	Surgical resection	Complete resolution	24	PMT	NA
Cheung et al. (22)	2006	46 M	Diffuse pain and multiple fractures	5	0.52 mmol/L (0.87–1.45 mmol/L)	111 octreotide scan, CT, MRI	Between 7th and 8th ribs on left	3 cm	Surgical resection	Complete resolution	12	PMTMCT	Smooth muscle actin, CD34 and FGF23 positive
Ferrari et al. (23)	2022	70 F	Diffuse pain, fatigue, and multiple fractures	NA	0.8 mg/dL (2.5–4.6 mg/dL)	68Ga-DOTATATE PET-CT	Under pleura near right eighth rib	3.0 × 1.5 × 1.0 cm	Surgical resection	Complete resolution	12	PMT	Vimentin positive
Gascón (24)	1999	51 F	Diffuse pain and fatigue	4	2.2 mg/dL (2.5–4.9 mg/dL)	Physical examination	Left breast	3 cm	Surgical resection	Complete resolution	3	Glandular sclerotic nodules and fibrous cystic disease of the breast	NA
Zhang et al. (25)	2023	27 M	Bone pain	NA	0.39 mmol/L (0.81–1.45 mmol/L)	68Ga-DOTATATE PET/CT	Nodule in upper lobe of the lung	0.7 cm	Surgical resection	Complete resolution	NA	PMT	NA
Zhang et al. (26)	2019	60 M	Diffuse pain and fatigue	4	NA	18F-FDG PET/CT, 68Ga-DOTATATE PET/CT	Soft tissues around umbilicus and lymph nodes in right axilla	NA	Surgical resection	Complete resolution	1.5	PMT and IGG4-related lymphadenopathy	IgG4 positive
Krishnappa et al. (27)	2019	50 M	Weakness in proximal lower limb muscles and hip pain	3	1.5 mg/dL	68Ga DOTATATE PET/CT, 18F-FDG PET/CT, 68Ga-DOTANOC PET/CT	Near spleen gate	2 × 1.4 cm	Surgical resection	Complete resolution	10	PMT	NA
Long et al. (28)	2020	61 F	Continuous fatigue and weakness	0.5	0.90 mmol/L (0.96–1.62 mmol/L)	68Ga-DOTATATE PET/CT, 99mTc	Right upper posterior mediastinum	1.9 × 1.2 cm	Surgical resection	Complete resolution	NA	Epithelioid hemangioendothelioma	CD34, CD31, and FLI-1 positive
Olivas-Mazón et al. (29)	2020	16 F	Abdominal pain, weight loss, and intermittent fever	0.25	1.3 mg/dL (2.5–4.9 mg/dL)	Physical examination, MRI, CT	Right upper abdomen	11.5 × 8 × 10.5 cm	Chemotherapy	The tumor shrank after chemotherapy	NA	Metastatic undifferentiated embryonic hepatic sarcoma	NA
Salim et al. (30)	2021	49 M	Rib fractures, generalized bone pain, and muscle weakness	3	1.6 mg/dL (2.5–4.9 mg/dL)	68Ga-DOTATATE PET-CT	Left upper lobe of lung	3.5 cm	Surgical resection	Complete resolution	36	HPT-PMT syndrome	NA
Todesco et al. (31)	2021	62 M	Weight loss and multiple fractures	NA	NA	68Ga-DOTATOC PET/CT, MRI	Left middle scalene muscle	1.6 cm	Surgical resection	Complete resolution	3	PMT	NA
Tsujimura et al. (32)	1996	54 M	Spasm, pain and weakness of thigh muscles on both sides	2	1.4 mg/dL (2.7–4.4 mg/dL)	Bone scan, CT	Lower posterior mediastinum	3 cm	Surgical resection	Complete resolution	1	PMTMCT	NA
Taylor et al. (33)	1988	57 M	Pain and discomfort in back, ribs, and legs and weakness in legs	5	1.8 mg/dL (2.5–4.9 mg/dL)	Bone scan and physical examination	Subcutaneous mass in right deltoid muscle area	2 cm	Surgical resection	Complete resolution	24	PMTMCT	NA
Ewain et al. (34)	2024	56 M	Progressive lower extremity weakness	4	0.28 mmol/L (0.74–1.52 mmol/L)	18F-FDG PET/CT, CT	Right pleura	3.0 x 2.0 cm	Surgical resection	Complete resolution	24	Epithelioid hemangioendothelioma	ERG positive

M, male; F, female; PMT, phosphaturic mesenchymal tumor; PMTMCT, phosphaturic mesenchymal tumor mixed connective tissue variant; NA, none; CT, computed tomography; MRI, magnetic resonance imaging; OCT, octreotide; PET/CT, positron emission tomography/computed tomography. The diagnostic delay time represents the time from the onset of symptoms to the diagnosis of TIO, measured in “years.” The follow-up time is measured in “months.” NA indicates that the data does not exist.

### 2.3 Data collection

We collected data on age, sex, clinical characteristics, diagnostic delay time, serum phosphate level, diagnostic method, tumor location and size, treatment, outcome, follow-up duration, histological diagnosis, and immunohistochemistry from 33 patients with soft-tissue TIO of the trunk. Diagnostic delay time was defined as the interval from symptom onset to the diagnosis of TIO.

### 2.4 Statistical analysis

SPSS version 23 software was used to analyze the data. Normality was evaluated using the Shapiro-Wilk test. The mean ± standard deviation was used to represent data that fit a normal distribution, whereas the median and interquartile range (IQR) were used to represent data that did not.

## 3 Results

Thirty-three cases of soft-tissue TIO of the trunk (excluding the present study) were identified, and the detailed clinical characteristics of these patients are presented in **Table 2**. The mean age of the patients was 49.7 ± 15.6 years, and the male-to-female ratio was 23:10. The clinical presentations included pain in 30 of 33 cases (91%), fractures in 7 of 33 cases (21%), and malaise in 16 of 33 cases (48%). The tumor involved bone in 8 cases (24.2%) and was limited to soft tissue in the remaining 25 cases. A diagnostic delay of more than two years was observed in 72.4% of cases. The mean preoperative serum phosphorus level was 0.48 ± 0.137 mmol/L (reference range: 0.81–1.45 mmol/L), and the median tumor size was 3 cm (IQR: 2–4.65 cm). Tumor localization methods included physical examination (14 cases), 68 Gallium-DOTA-Tyr3-octreotate positron emission tomography - computed tomography (^68^Ga-DOTATATE PET/CT) (8 cases), MRI (13 cases), computed tomography [(CT), 10 cases], bone scan (5 cases), PET-CT (4 cases), ^18^F-FDG PET/CT (4 cases), and ultrasound (2 cases).

For treatment, 32 patients underwent surgical resection, while one patient with metastatic undifferentiated embryonal hepatic sarcoma received chemotherapy. Clinical symptoms gradually improved, and biochemical indices returned to normal postoperatively in 31 patients. However, one patient died at the age of 18 years due to postoperative malignant transformation with multiple metastases. The median follow-up time of the 33 patients was 10 months (IQR: 3–24 months). Postoperative pathology in 72.7% of patients with TIO was reported as PMT or phosphaturic mesenchymal tissue tumor mixed connective tissue subtype (PMTMCT). The remaining tumor pathologies included two cases each of sarcoma, osteosarcoma, angioepithelial cell tumor, epithelioid hemangioendothelioma, and one case of fibrocystic breast nodule. Immunohistochemistry was missing in 23 of the 33 patients. The immunohistochemical results of the remaining patients demonstrated three cases of FGF23 positivity, three cases of CD34 positivity, two cases of vimentin positivity, and one case each of SSTR2A, CD31, FLI-1, ERG, P53, IgG4, and SMA positivity.

## 4 Discussion

Herein, we report a case of TIO caused by a soft tissue tumor in the back. We also reviewed cases of TIO that occurred in the trunk in the literature. Our patient presented with generalized bone pain and weakness in both lower extremities. Laboratory tests indicated hypophosphatemia, elevated simultaneous urinary phosphorus, and elevated alkaline phosphatase. Whole-body DR scans showed “double depression” in some vertebrae, and lateral head films also revealed blurred trabeculae and other characteristic changes of osteomalacia. Hypophosphatemic osteomalacia was considered. A mass was found in the back during physical examination, and its location and relationship to surrounding tissues were further evaluated using MRI. Based on the above clinical manifestations and laboratory tests, TIO was considered. Owing to the superficial location of the mass, we performed a direct resection. On the third day after surgery, the patient’s blood phosphorus level returned to normal, and the symptoms of bone pain and fatigue were significantly alleviated.

Postoperative pathology suggested a PMT. Immunohistochemistry showed spindle cells that were positive for Vim, CD56, Ki-67 (with a hotspot area of approximately 10%), FGF23, SSTR2A, and SATB2, but negative for CD34.

TIO is a rare disease, with an estimated prevalence of 0.70 per 100,000 population in one epidemiological study (35) and fewer than 1,000 cases reported worldwide (36). A study by Bosman et al. (37), which included 895 cases of TIO, showed that tumors can occur in any part of the body, with the most common locations being the lower extremities, head and neck, pelvis, and trunk in that order. Jan De Beur et al. (38) reported that TIO of the trunk accounted for only 14% of all TIO cases. Clinically, TIO should be considered in patients presenting with chronic bone pain, fatigue and weakness, and fragility fractures, and refinement of serum phosphate testing is recommended. Once biochemical results support a diagnosis of TIO, a combination of functional and anatomical imaging is required to localize the tumor. Unlike bone tumors, some soft-tissue tumors are relatively superficial, and careful physical examination is valuable in detecting these tumors. In this study, tumors were detected by physical examination in 42% of cases, which is higher than the 32.4% reported in the systematic review by Bosman et al. (37), suggesting that physical examination is particularly important in identifying superficial soft-tissue tumors. Primary care hospitals should identify suspected soft-tissue tumors during physical examination and evaluate their relationship to adjacent tissues using MRI or CT. If the physical examination is negative, then functional imaging should be performed to identify the approximate location of the tumor. Conventional Octreoscan SPECT/CT imaging (e.g., 99mTc-octreotide SPECT/CT imaging or 111In-octreotide SPECT imaging) improves tumor detection (39, 40), but its spatial resolution is limited. ^68^Ga-DOTATATE, a ^68^Ga-labeled somatostatin analog, binds to the PMT-expressed SSTR to localize tumors (41). In our study, ^68^Ga-DOTATATE PET/CT successfully localized tumors in 24% of patients with trunk TIO. For lesions in deep or anatomically complex areas, ^68^Ga-DOTATATE PET/CT is particularly advantageous (16). Hou et al. (42) showed that ^68^Ga-DOTATATE-PET/CT had a sensitivity of 94.7%, which was higher than the sensitivity of Octreoscan SPECT/CT (86.3%) (43) and ^18^FDG-PET/CT (80.0%) (44). Global TIO guidelines recommend ^68^Ga-DOTATATE PET/CT, ^68^Ga-DOTANOC PET/CT, and ^68^Ga-DOTATOC PET/CT as first-line functional imaging, followed by octreotide scans (99mTc- or 111In-pentetreotide scans) or FDG-PET (1).

Complete surgical resection is the preferred treatment option for TIO (45). In this study, 96.9% of patients experienced postoperative remission of biochemical markers and clinical symptoms with no recurrence during the follow-up period. Meanwhile, 3.1% developed metastatic tumors postoperatively, and 3.0% had metastases present at the time of initial diagnosis, for which chemotherapy was the treatment of choice. Studies have shown that limb soft-tissue tumors yield better surgical outcomes than intraosseous tumors associated with TIO (46). For patients with inoperable or metastatic TIO, treatment typically involves oral phosphate combined with osteotriol, although long-term use can lead to secondary hyperparathyroidism or renal calcification (47). Novel therapies, such as the anti-FGF23 monoclonal antibody, Burosumab, significantly improve biochemical markers and quality of life by restoring renal phosphorus reabsorption and vitamin D metabolism through FGF23 inhibition (48, 49). Crotti et al. (48) reported a case of a European patient who, after three unsuccessful surgeries, was treated with Burosumab for two years, resulting in significant improvement in both pain and blood phosphorus levels. However, Burosumab does not inhibit tumor progression, so patients should continue to pursue surgical resection while undergoing treatment. In cases where patients do not improve after surgery, clinicians should consider the possibility of multifocal tumors or malignant transformation. This study suggests that although most of the tumors causing TIO are benign, long-term follow-up remains essential after surgery.

Patients with TIO exhibit diverse tumor pathology, with most pathology reports identifying PMT or PMTMCT. The concept of PMT was first introduced by Weidner and Cruz (50) in 1987, who classified four morphological subtypes: PMTMCT, osteoblastoma-like, non-ossifying fibroma-like, and ossifying fibroma-like tumors (51). In 2004, Folpe et al. (52) further clarified that most PMTs can be categorized as the mixed connective tissue subtype. PMTs exhibit varied histological features. Tumor cells typically range from small round cells to short, spindle-shaped, fibroblast-like cells. These tumors are usually accompanied by numerous thick- and thin-walled small blood vessels of mesenchymal origin with diffuse infiltration. In some cases, osteoid and cartilaginous stroma, gravel-like calcifications, and osteoblastic giant cells are also observed (53). Due to its lack of specific histological features and considerable morphological overlap with other soft-tissue tumors, PMT is frequently misdiagnosed as hemangioma, ossifying fibroma, osteosarcoma, or chondrosarcoma (53). Immunohistochemistry aids in determining tumor pathology; however, studies on the immunophenotype of PMT remain limited both locally and internationally. According to the literature, PMTs express various immunohistochemical markers to differing extents, including FGF23, vimentin, NES, CD56, CD68, SMA, CD34, Bcl-2, D2-40, and CD99. Reported positivity rates include 88.4% for FGF23 (53), 90% for SATB2, and 79% for SSTR2A (54). In our cases, immunohistochemical staining was positive for FGF23, SSTR2A, and SATB2. Among the trunk cases analyzed, all three showed positive FGF23 immunohistochemical staining (3/3; 100%), and one case was positive for SSTR2A. Co-expression of FGF23 and SSTR2A is highly sensitive for diagnosing PMT but lacks specificity; however, negative results can effectively exclude PMT (55–57). A recent study by Agaimy et al. (54) further reported frequent expression of SATB2, CD56, and ETS-related gene (ERG) in PMT. Other supportive markers, including NSE, CD99, Bcl-2, and D2-40, are commonly positive, while SMA and CD34 may show focal expression (58). In contrast, S-100 protein, junctional proteins, and similar markers are typically not expressed (59). According to Chatterjee et al., SATB2+/ERG+/CD56+/S-100-/STAT6- is an immunophenotype that assists in distinguishing PMT from numerous histologic mimics (60). Compared with the immunophenotypes of other patients with TIO in the trunk and soft tissue, the immunophenotype of our patient (SATB2+/SSTR2A+/CD56+/FGF23+) was relatively complete, which is also a strong basis for diagnosis.

For patients with generalized bone pain and fatigue, completing the blood phosphorus test in a timely manner and considering the possibility of TIO are necessary. A comprehensive and detailed physical examination of the whole body is the simplest and most necessary localization method for diagnosis (61). For patients who are highly suspected of having TIO and superficial soft tissue masses, the masses can be completely removed. This is both a diagnostic and therapeutic method. Typical pathological features and comprehensive immunohistochemical analysis can help with the final diagnosis.

### 4.1 Study limitations

A key limitation of this study is the absence of FGF23 measurement, as it is not routinely tested at the authors’ hospital. Consequently, we were unable to observe its dynamic changes throughout all stages of the disease. In addition, no genetic testing was conducted on the patients and no further in-depth research was carried out.

## 5 Conclusion

We report a soft-tissue PMT in the trunk that resulted in TIO. Based on our case and a review of the relevant literature, we highlight three key points: First, complex imaging is not always necessary for patients with TIO, and the role of physical examination in localizing superficial soft-tissue PMTs should not be underestimated. Second, clinicians should remain vigilant for tumor-associated hypophosphatemic osteomalacia in patients presenting with unexplained bone pain and hypophosphatemia and should promptly conduct appropriate examinations to avoid missed diagnoses. Third, although the prognosis following PMT resection is generally favorable, long-term follow-up is essential to detect multifocal disease or malignant transformation at an early stage.

## Data Availability

The original contributions presented in the study are included in the article/supplementary material. Further inquiries can be directed to the corresponding authors.
